# Island biogeography theory provides a plausible explanation for why larger vertebrates and taller humans have more diverse gut microbiomes

**DOI:** 10.1093/ismejo/wrae114

**Published:** 2024-06-21

**Authors:** Katherine Ramos Sarmiento, Alex Carr, Christian Diener, Kenneth J Locey, Sean M Gibbons

**Affiliations:** Institute for Systems Biology, Seattle, WA 98109, United States; Institute for Systems Biology, Seattle, WA 98109, United States; Molecular Engineering Graduate Program, University of Washington, Seattle, WA 98195, United States; Institute for Systems Biology, Seattle, WA 98109, United States; Diagnostic and Research Institute of Hygiene, Microbiology and Environmental Medicine, Medical University of Graz, 8010 Graz, Austria; Center for Quality, Safety & Value Analytics, Rush University Medical Center, Chicago, IL 60612, United States; Institute for Systems Biology, Seattle, WA 98109, United States; Molecular Engineering Graduate Program, University of Washington, Seattle, WA 98195, United States; Department of Bioengineering, University of Washington, Seattle, WA 98195, United States; Department of Genome Sciences, University of Washington, Seattle, WA 98195, United States; Science Institute, University of Washington, Seattle, WA 98195, United States

**Keywords:** gut microbiome, alpha diversity, 16S, island biogeography theory, macroecological scaling, body size, height, CDI

## Abstract

Prior work has shown a positive scaling relationship between vertebrate body size, human height, and gut microbiome alpha diversity. This observation mirrors commonly observed species area relationships (SARs) in many other ecosystems. Here, we expand these observations to several large datasets, showing that this size–diversity scaling relationship is independent of relevant covariates, like diet, body mass index, age, sex, bowel movement frequency, antibiotic usage, and cardiometabolic health markers. Island biogeography theory (IBT), which predicts that larger islands tend to harbor greater species diversity through neutral demographic processes, provides a simple mechanism for positive SARs. Using a gut-adapted IBT model, we demonstrated that increasing the length of a flow-through ecosystem led to increased species diversity, closely matching our empirical observations. We delve into the possible clinical implications of these SARs in the American Gut cohort. Consistent with prior observations that lower alpha diversity is a risk factor for *Clostridioides difficile* infection (CDI), we found that individuals who reported a history of CDI were shorter than those who did not and that this relationship was mediated by alpha diversity. We observed that vegetable consumption had a much stronger association with CDI history, which was also partially mediated by alpha diversity. In summary, we find that the positive scaling observed between body size and gut alpha diversity can be plausibly explained by a gut-adapted IBT model, may be related to CDI risk, and vegetable intake appears to independently mitigate this risk, although additional work is needed to validate the potential disease risk implications.

## Introduction

From birth, vertebrate animals are colonized by a diverse community of commensal microbiota that they carry throughout their lives [1]. The vast majority of these commensal microbes reside in the gastrointestinal tract [2]. The human gut microbiota has an enormous impact on our phenotype [3], with almost half of the metabolites circulating in blood significantly associated with cross-sectional variation in the ecological composition of the gut microbiome [4, 5]. One of the key ecosystem functions that the gut microbiota provides to its host is resistance to enteric bacterial pathogens [6]. Niche saturation or nutrient competition are commonly invoked mechanisms for how the microbiota excludes invaders [6, 7]. Specifically, species-diverse commensal communities are more apt to saturate available metabolic niches so that an invasive pathogen is less likely to colonize, outcompete commensals, and cause disease [6–8]. Though many determinants of gut microbiome alpha diversity (i.e. taxon richness and/or evenness in a given sample) are known, including diet, intestinal transit time, and antibiotic treatment, much of the variation in gut alpha diversity remains unexplained [9–12].

Vertebrate body size, which varies over six orders of magnitude, has been shown to be positively associated with gut microbiome alpha diversity, indicating that larger animals with larger guts harbor more species [13]. This prior work used an older method for quantifying alpha diversity that involved rapidly transitioning single-stranded DNA from 16S rRNA gene amplicons from warmer to colder temperatures so that single strands quickly fold into unique shapes that are determined by their primary sequence. These unique, negatively charged single-stranded DNA strands migrate at different rates through a capillary tube during electrophoresis, with each band representing a different taxon, and laser-induced fluorescence detection allows for band/taxon quantification [13, 14]. Similarly, recent work from a large human cohort, using 16S rRNA gene amplicon sequencing, showed a positive association between height and gut microbiome alpha diversity [15]. We were curious to understand the mechanism(s) underlying these scaling patterns.

Body size versus gut microbiome diversity scaling patterns mirror similar species area relationships (SARs) seen in other ecosystems, where larger geographic areas correspond to more observed taxa [16–18]. The mechanisms underlying SAR-like behavior in the gut, and the potential clinical consequences of this scaling, have yet to be explored. Prior literature has shown that simple neutral processes are able to replicate certain features of gut microbiome community dynamics [19, 20]*.* Island biogeography theory (IBT), a classic neutral model of species immigration/emigration, birth/death, and speciation/extinction, predicts a positive SAR [21]. Specifically, larger islands tend to harbor more individuals, which ultimately gives rise to a larger number of coexisting species [21]. We hypothesized that IBT may explain the size–diversity relationships observed across vertebrates and within humans. To further explore this hypothesis, we built an individual-based model (IBM) that approximates IBT in the gut, allowing for variation in system length, with immigration, emigration, reproduction, and a unidirectional flow-through system. We simulated length ranges that approximate the scaling of vertebrate body sizes and human height variation, with residence time (i.e. the product of the flow rate and the length of the system) as our proxy for area, and compared these simulation results to our empirical observations.

The potential clinical significance of this scaling pattern lies in the fact that lower gut microbiome alpha diversity has been associated with greater susceptibility to enteric infections [8, 22]. As mentioned above, the mechanism for this relationship between infection risk and diversity is likely related to the niche saturation hypothesis, which posits that a more diverse commensal microbiota protects the host from enteric pathogens through competitive exclusion [7, 23, 24]. *Clostridioides difficile*, an opportunistic enteric pathogen, is the most common form of hospital-acquired colitis in the USA, and susceptibility to this disease is strongly related to common diversity-reducing disruptions to the commensal gut microbiota, such as antibiotic treatment or diarrhea [8, 25–27]*. Clostridioides difficile* infections (CDIs) are initially treated with oral antibiotics, and recurrent illness is common, especially in those starting out with lower gut microbiome alpha diversity [8, 28, 29]. We hypothesized that, due to the scaling between height and gut microbiome alpha diversity, individuals who report a history of enteric infection may be slightly shorter, on average, than those who do not.

Overall, we demonstrate a consistent scaling between body size and gut microbiome alpha diversity across vertebrates and human populations. We find that this association is independent of many potential confounders, like diet, bowel movement frequency (BMF), body mass index (BMI), age, and sex. We provide a plausible mechanistic hypothesis for this size–diversity scaling, based on a gut-adapted IBT. Finally, we show evidence that this scaling may be relevant to human health, although additional validation will be necessary to assess the clinical relevance of this observation.

## Materials and methods

### Published datasets

In order to investigate the relationship between vertebrate body size and gut microbiome alpha diversity, we used three independent datasets: Godon *et al*. 2016, Song *et al*. 2020, and Groussin *et al*. 2017 [13, 30, 31].

The Godon *et al.* 2016 dataset included pre-calculated Simpson’s diversity [13], derived from capillary electrophoresis single-strand conformation polymorphism (CE-SSCP) fluorescence patterns of DNA amplicons from the V3 region of the 16S rRNA gene [32], and body mass for 71 vertebrate species, where bacterial diversity was assessed by extracting DNA from feces. These samples were obtained from captive or domesticated vertebrates in France from either zoos, farms, aquariums, recreational farms, or individual keepers. The metadata for these samples included information about the individual’s diet type and mass, which were curated from literature for the smaller species or provided by the breeders for the larger species.

Song *et al.* 2020 included 16S rRNA gene amplicon sequencing data derived from 1373 samples from 164 vertebrate species’ fecal samples, intestinal contents, or aspirations of the large intestine. Samples that showed signs of contamination from soil or environmental bacteria, from juveniles/newborn individuals, and from diseased individuals were removed. Moreover, duplicate samples from the same individual were removed and samples were included only if the host species had been sampled at least twice. ASVs that were not of bacterial origin were removed. Samples with no information on country of origin and/or information on the preservative used were removed. This dataset included fecal samples from vertebrates living in wild and captive populations. The metadata for this dataset included the body mass and diet type of the vertebrate. We restricted our vertebrate analyses to carnivores and herbivores, removing insectivores and omnivores for clearer estimation of the effect of diet by making it a binomial categorical variable.

Groussin *et al.* 2017 includes 16S rRNA gene amplicon sequencing data derived from fecal samples from 33 mammalian species. The operational taxonomic unit (OTU) table was downloaded from Groussin’s publicly available GitHub repository “MammalianGuts” and had been processed as described in Groussin *et al.* 2017 (https://github.com/mgroussi/MammalianGuts). Animal masses were not included from this study, but average masses for each species were manually curated from literature and from the AnimalTraits Database [33–57], and these masses are now deposited in the GitHub repository for this paper (see data availability section). These samples were taken from both wild and captive populations of vertebrates.

The data collected from the Arivale cohort included height, BMI, sex, BMF, vegetable consumption frequency, and 16S rRNA gene amplicon data derived from stool samples. We used the mbtools workflow (https://github.com/gibbons-lab/mbtools) to denoise the 16S rRNA gene data. Moreover, training error models and removal of chimeric reads using DADA2 was done separately for each sequencing run to generate amplicon sequence variants (ASVs) for each sample [58].

The American Gut cohort consisted of self-selected adults participating in a citizen-science program, primarily from the USA, the UK, and Australia [9, 59]. We downloaded the American Gut data from figshare (https://doi.org/10.6084/m9.figshare.6137192.v1), with the metadata including self-reported height, weight, sex, age, BMF, CDI history, and vegetable intake frequency. We used the self-reported height and weight to calculate BMI for each participant. The sub operational taxonomic unit (sOTU) table was generated by the original American Gut authors using Deblur [60], and all reads were trimmed to a length of 125 nucleotides. Moreover, because samples had been sent via mail at room temperature, samples with obvious bacterial blooms that occurred in the sample tubes were removed. Samples were rarefied to 1250 reads and samples with <1250 sequences were not included in the sOTU table. Subsequent filtering on the data was performed in order to avoid including erroneous self-reported data: we filtered out individuals who were <18 years old; we removed samples from participants who reported a height >244 cm or a height <122 cm; and we removed participants who reported a weight ≥300 kg.

The Arivale and American Gut cohort metadata included equivalent information about each individual’s age, sex, height, and BMI. However, the way that BMF was recorded for the American Gut cohort was different from how it was recorded for the Arivale cohort. In the Arivale cohort questionnaire, participants were prompted to respond to: “I have bowel movements” with the options: “2 or fewer times per week,” “3–6 times per week,” “1–3 times daily,” and “4+ times daily.” The American Gut bowel questionnaire prompted individuals to respond to: “How many times do you have a bowel movement in an average day?”, where individuals could choose from the options: “Less than one,” “one,” “two,” “three,” “four,” or “five or more.” In addition, dietary reporting questions were slightly different across cohorts. American Gut cohort participants were prompted to report their weekly dietary intake of vegetables by responding to the prompt: “In an average week, how often do you consume at least 2–3 servings of vegetables, including potatoes in a day? (1 serving = ½ cup vegetables/potatoes; 1 cup leafy raw vegetables)” and chose from the following responses: “a. Never,” “b. Rarely (less than once/week),” “c. Occasionally (1–2 times/week),” “Regularly (3–5 times/week),” and “e. Daily.” Individuals in the Arivale cohort were prompted to report their weekly dietary intake of vegetables by responding to the prompt: “How many servings of vegetables (including juice) do you have each day? (1 serving = 1 cup raw/leafy vegetables, ½ cup cooked vegetables, or ½ cup vegetable juice)” and chose from the following responses: “Zero/less than 1 per day,” “1,” “2–3,” “4–5,” or “6 or more.” We standardized BMI, age, log-Simpson’s diversity, and log-height by calculating their *Z*-scores ($Z=\left(x-\mathrm{mean}(x)\right)/\mathrm{std}(x)$) within each cohort prior to statistical analyses. We categorized individuals in each human cohort into “high” and “low” vegetable consumption groups when binning the two groups in separate regressions (Fig. 2C–D). Individuals in both the American Gut and Arivale cohorts were categorized into the high vegetable consumption group if they reported at least 1 serving of vegetables a day, with anyone reporting less being included in the low vegetable consumption group.

### Sequencing data processing and diversity calculations

We used Qiime 2-2022.8 to rarefy our ASV and OTU tables and to compute the Simpson’s diversity metric across samples. The rarefaction depth (i.e. the minimum sampling depth) for each dataset was as follows: Song *et al.* 2020 (sampling depth: 5035), Groussin *et al.* 2017 (sampling depth: 1266), Arivale cohort (sampling depth: 13 700), and American Gut cohort (sampling depth: 1250). Because Qiime 2-2022.8 returned Simpson’s diversity as 1 − *D*, we converted the Simpson’s diversity returned by Qiime (Q_simpson) values to Simpson’s diversity (1/*D*) using the formula 1/*D* = 1/(1 − Q_simpson).

### Simulations

All of our simulated digestive tracts had identical parameters for the shape of the lognormal meta-community population distribution (lgp = 0.9999), reproduction rate (*r* = 0.01), and immigration rate (im = 25). Our decision to sample from a lognormal population distribution was meant to approximate the heavy-tailed species abundance distribution that has been observed in the gut microbiome. Furthermore, lognormal SADs have been predicted by Hubbell’s unified neutral theory of biodiversity [61] and have been successfully applied to describe real-life SADs of both microbial and non-microbial communities [61–65]. For each individual in our simulated system, a species ID (represented by an integer) and an *x*-coordinate (*x*-coordinate = 1) were assigned during the immigration process. For each individual simulation, the length of the system was randomly determined using the numpy.random module (version 1.23.3). The reproduction rate determined the percentage of the population randomly selected for reproduction per timestep. In our simulations, 1% of the population were randomly selected for reproduction and the resulting progeny inherited the species ID and current *x*-coordinate of their mother cell. The immigration rate determined the number of new individuals sampled from the meta-community that entered the system at each time step, and individual species IDs were determined according to a lognormal distribution of integers (i.e. there were more 1 s than 2 s, more 2 s than 3 s). At each time step, each individual moved one unit down the length of the system relative to their current position during a process defined as “flow.” If an individual’s *x*-coordinate was larger than the length of the system, their species ID and *x*-coordinate were deleted from the model. Apart from receiving a unique ID, each species was functionally identical (i.e. this is a purely neutral model).

Once these parameters were determined, a simulation was initialized, in which the processes—immigration, reproduction, and flow—were carried out at each time step. The order in which these processes took place was randomized for each time step to remove systematic biases. Simulations continued iterating through these processes until the number of individuals and the number of species reached stationarity, which was determined using an Augmented Dickey–Fuller test using the statsmodels package after at least 1000 time steps had been completed (version 0.13.5) [66]. When stationarity was reached, the simulation stopped and the steady-state Simpson’s diversity was calculated based on the individuals and species in the system and printed to a CSV file at a location which can be defined by the user in main.py (see code availability section).

### Statistical analyses

Ordinary Least Squares (OLS) regression (statsmodels 0.13.2) was used to test the association between vertebrate body size and gut microbiome alpha diversity, using the formula: log-Simpson’s diversity ~ log-mass (kg). We used the numpy package to log transform the Simpson’s diversity (1/*D*) and the mass (kg) for all vertebrate datasets. To further investigate the role of diet and the interaction between diet and body mass, we also carried out another OLS regression using the formula: log-Simpson’s diversity ~ log-mass (kg) + diet + diet:log-mass (kg). Similarly, the association between height and gut microbiome alpha diversity in the Arivale and the American Gut cohorts was tested using OLS regression, first with a univariate model, with the formula: log-Simpson’s diversity ~ log-height, and secondly with a multivariate model with the formula: log-Simpson’s diversity ~ log-height + age + sex + vegetable consumption + BMI + BMF. In order to assess the significance of height in the OLS models, we used an analysis of variance (ANOVA) *F*-test (statsmodels 0.13.2) to compare a reduced model with the formula: Simpson’s diversity ~ age + sex + vegetable + BMI + BMF to the full model, which includes height, described above. For our health-adjusted analysis in the Arivale dataset, we added multiple health markers measured from participant blood plasma as covariates in our OLS regression, including low-density lipoprotein (LDL) cholesterol, C-reactive protein (CRP), and hemoglobin A1C (HbA1C), excluding individuals who reported antibiotic usage within the last 3 months. We used the following formula: log-Simpson’s diversity ~ log-height + age + sex + vegetable consumption + BMI + BMF + height + vegetable consumption: height + LDL-cholesterol + CRP + HbA1C. In the American Gut cohort, we performed a Welch’s *t*-test (scipy 1.9.1) with Bonferroni correction (correcting for three tests) to assess the mean difference in Simpson’s diversity between individuals with and without a history of CDI, as well as within the groups “low vegetable intake” and “high vegetable intake.” Similarly, we used a Welch’s *t*-test with Bonferroni correction correcting for three comparisons per hypothesis in order to compare the mean heights of individuals with and without a history of CDI in the entire cohort and within the groups: low vegetable intake and high vegetable intake. We defined individuals with high vegetable intake as individuals who ate vegetables at least once a day. Individuals who ate their vegetables less than once a day were placed in the low vegetable intake category. We conducted our causal mediation analyses in R, using the “mediation” package (version 4.5.0) [67], with height or diet as the treatment, alpha diversity as the mediator, and CDI history as the response. The significance threshold for all tests was set at *P* < .05.

### Data and code availability

All code, notebooks, and intermediate data files related to data analysis and IBM simulations can both be found in the following GitHub repository: https://github.com/Gibbons-Lab/IBT-and-the-Gut-Microbiome. Raw data from the Godon *et al.* 2016, Song *et al.* 2020, and Groussin *et al.* 2017 studies can be accessed in the original papers, or above in the “*Published datasets*” section [13, 31, 68]. The American Gut dataset was downloaded from figshare and can be found at the following link: https://figshare.com/articles/dataset/American_Gut_Project_fecal_sOTU_counts_table/6137192. Qualified researchers can access the full Arivale deidentified dataset supporting the findings in this study for research purposes through signing of a data use agreement. Requests to access the Arivale data can be made at data-access@isbscience.org and will be responded to within seven business days.

## Results

### Positive scaling between vertebrate body size and gut microbiome alpha diversity

**Figure 1:**
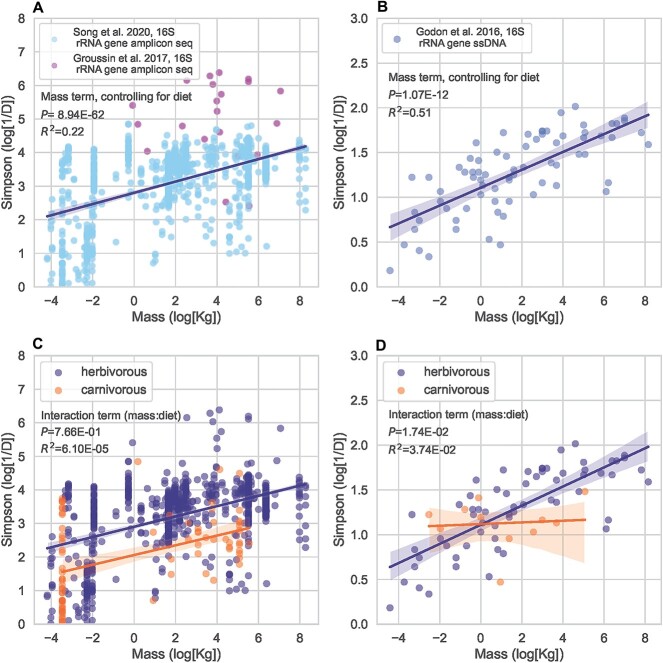
Relationship between body size and gut microbiome Simpson’s diversity across vertebrates. (A) Log-body mass and log-Simpson’s diversity are positively associated across two independent 16S rRNA gene amplicon sequencing datasets measuring gut alpha diversity among vertebrates. (B) A similar result emerges from a CE-SSCP dataset. (C) Here, we show OLS regression analysis (formula: log-Simpson’s diversity ~ log-mass + diet + diet:log-mass) of the Song *et al*. 2020 and Groussin *et al*. 2017 datasets including diet and a diet:log-mass interaction effect. (D) Here, we show OLS regression analysis (formula: log-Simpson’s diversity ~ log-mass + diet + diet:log-mass) including diet and a diet:log-mass interaction effect.

We re-analyzed the Groussin *et al.* 2017 (*n* = 22) and Song *et al.* 2020 (*n* = 967) datasets (Fig. 1A and 1C), along with the Godon *et al.* 2016 dataset (*n* = 73, Fig. 1B and 1D), and observed a consistent, positive association between log-Simpson’s diversity and log-mass across all three studies (Fig. 1). OLS regression analysis (log-Simpson’s diversity ~ log-mass) yielded an *R*^2^ = .28, *P* < 10^−6^  in the Groussin *et al.* 2017 and Song *et al.* 2020 datasets, and yielded an *R*^2^ = 0.52, *P* < 10^−6^ in the Godon et al. 2016 dataset.

**Figure 2:**
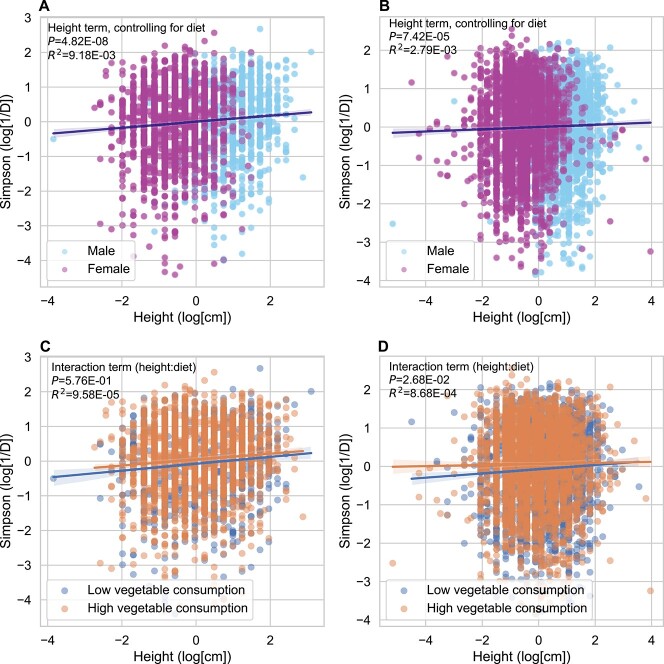
Relationship between height and gut microbiome Simpson’s diversity across humans. Both figures displayed are on log–log axes, displaying Simpson’s diversity versus height in (A) the Arivale cohort (*n* = 3063) and in (B) the American Gut cohort (*n* = 5516). Both plots show a similar trend where log-height is positively associated with log-Simpson’s diversity. OLS regressions used the formula: log-Simpson’s diversity~ sex + age + BMI + BMF + vegetable consumption + log-height. Adding an interaction effect to our OLS regressions (log-Simpson’s diversity~ sex + age + BMI + BMF + vegetable consumption + log-height:vegetable consumption + log-height) did not yield a significant interaction effect in the (C) Arivale cohort (*P* = .576), but did in the (D) American Gut cohort (*P* = 2.68 ∙ 10^−2^).

We added diet (herbivore vs. carnivore) as a covariate to this regression analysis (log-Simpson’s diversity ~ log-mass + diet). Log-body mass remained significantly associated with log-Simpson’s diversity in both cohorts. The Godon *et al.* dataset yielded an *R*^2^ = 0.51, *P* = 1.07 ∙ 10^−12^ for the mass term and an *R*^2^ = 6.70 ∙ 10^−3^, *P* = .323 for the diet term. The Song *et al.* 2020 and Groussin *et al.* 2017 yielded an *R*^2^ = 0.22, *P* < 10^−6^ for the mass term and an *R*^2^ = 0.05, *P* < 10^−6^ for the diet term.

We added an interaction effect term between diet and log-mass in the OLS regression models to assess the impact of diet on the observed body-size scaling (log-Simpson’s diversity ~ log-mass + diet + diet:log-mass; Fig. 1C–D). The diet:log-mass interaction term was statistically significant in the Godon *et al.* 2016 dataset (*P* = .0174), but not in the combined Groussin *et al.* 2016 and Song *et al.* 2020 datasets (*P* = .770). If we use the Fisher’s method to combine the interaction term *P* values, we do not find evidence for a consistent effect across datasets (combined *P* = .0710). Overall, the effect of vertebrate body mass on diversity is significant, independent of diet, though results were mixed in the presence of an interaction term between mass and diet.

### Relationship between human height and gut microbiome alpha diversity is robust to inclusion of relevant covariates

We found that human height and gut microbiome alpha diversity were positively associated across two large, independent human cohorts. We used data from the Arivale cohort (*n* = 3063) and the American Gut cohort (*n* = 5516) to compare log-Simpson’s diversity versus log-height (Fig. 2). The Arivale cohort consisted of self-selected American adults who had enrolled in a scientific wellness program primarily from the Pacific West of the United States, and the American Gut cohort consisted of self-selected adults participating in a citizen-science program, primarily from the USA, the UK, and Australia [9, 59].

Because several demographic variables are known to affect gut microbiome diversity, we ran OLS regressions within each cohort, with log-height, age, sex, BMI, BMF, vegetable consumption, and vegetable consumption:log-height interaction terms (log-Simpson ~ age + sex + BMI + BMF + vegetable consumption + log-height + vegetable consumption:log-height). Consistent with the literature, we found that in both cohorts, being male, having a higher BMF, and a higher BMI were all negatively associated with log-Simpson’s diversity, while height, age, and vegetable consumption were all positively associated with log-Simpson’s diversity (Table 1). Log-height was positively associated with Simpson’s diversity in the presence and in the absence of the covariates listed above (Table 1). In addition, an ANOVA comparing the univariate model to an intercept-only model found that there was a significantly higher fraction of variance explained by a model including only height across both cohorts (*F*-test *P* = 1.31 ∙ 10^−6^ for the Arivale cohort; *P* = 3.11 ∙ 10^−2^ for the American Gut cohort; Table 1). Furthermore, an ANOVA, comparing a reduced model without height (covariates only) versus a full model including height, showed that there was a significantly higher fraction of variance explained by the full model across both cohorts (*F*-test *P* < 10^−6^ for the Arivale cohort; *P* = 3.36 ∙ 10^−5^ for the American Gut cohort; Table 1). A paired *Z*-test determined that there was no significant difference in height $\beta$ coefficients between the univariate and multivariate models for the Arivale dataset (*Z* = 1.25, *P* = .211). There was a significant difference between the univariate and multivariate models for the American Gut dataset (*Z* = 2.69, *P* = .00716), but we did not think this difference in coefficients was interpretable or meaningful given the small magnitude of the coefficients as well as the collinearities among the covariates (e.g. sex and height). We did not find evidence of a significant interaction effect between height and vegetable intake in the Arivale dataset, but we did in the American Gut dataset (*P* = .576 and *P* = .0268, respectively; Table 1).

**Table 1:** Ordinary Least Squares (OLS) regression shows height’s association with Simpson’s diversity is robust to inclusion of several relevant covariates.

Cohort	Covariate	*R* ^2^	*P* (covariate)	Beta	Model	*R* ^2^ (model)	*F*	*P* (*F*)
Arivale	Sex[T.M]	0.0013	4.15E-02	−0.1058	Multivariate	0.0628	15.12	2.92E-07
Arivale	Age	0.0058	1.53E-05	0.0762	Multivariate	0.0628	15.12	2.92E-07
Arivale	BMI	0.0317	6.94E-24	−0.1804	Multivariate	0.0628	15.12	2.92E-07
Arivale	BMF	0.0097	2.05E-08	−0.1831	Multivariate	0.0628	15.12	2.92E-07
Arivale	Vegetable frequency	0.0014	3.26E-02	0.0440	Multivariate	0.0628	15.12	2.92E-07
Arivale	Height	0.0023	6.30E-03	0.1670	Multivariate	0.0628	15.12	2.92E-07
Arivale	Height:vegetable frequency	0.0001	5.76E-01	−0.0113	Multivariate	0.0628	15.12	2.92E-07
Arivale	Height		1.31E-06	0.0873	Univariate	0.0076	23.50	1.31E-06
American Gut	Sex[T.M]	0.0009	2.18E-02	−0.0855	Multivariate	0.0246	10.32	3.36E-05
American Gut	Age	0.0062	3.47E-09	0.0806	Multivariate	0.0246	10.32	3.36E-05
American Gut	BMI	0.0005	9.91E-02	−0.0227	Multivariate	0.0246	10.32	3.36E-05
American Gut	BMF	0.0081	1.48E-11	−0.0959	Multivariate	0.0246	10.32	3.36E-05
American Gut	Vegetable frequency	0.0051	7.95E-08	0.0907	Multivariate	0.0246	10.32	3.36E-05
American Gut	Height	0.0017	1.77E-03	0.2306	Multivariate	0.0246	10.32	3.36E-05
American Gut	Height:vegetable frequency	0.0009	2.68E-02	−0.0363	Multivariate	0.0246	10.32	3.36E-05
American Gut	Height		3.11E-02	0.0290	Univariate	0.0008	4.65	3.11E-02

Height retains its significant association with diversity in the presence and in the absence of covariates across both cohorts. *F*-Tests, comparing a reduced model without height (including all covariates) to a full model including height, indicates that height provides a significant increase in the variance explained by the full model.

We followed up these regressions with a health-adjusted analysis in the Arivale cohort, in order to exclude the possibility that these patterns were driven by differences in host health status. We excluded individuals who reported antibiotic use in the last 3 months and we added the following health markers as covariates to the multivariate model: LDL cholesterol, CRP, and HbA1C (log-Simpson ~ age + sex + BMI + BMF + vegetable consumption + log-height + vegetable consumption:log-height + LDL + CRP + HbA1C). In this health-adjusted analysis, height retained its significance (*P* = 3.76 ∙ 10^−3^) and its beta-coefficient was similar (*β* = 0.1920) when compared to the non-health-adjusted multivariate model (*β* = 0.1670). In summary, the relationship between height and diversity is independent of these covariates across two large, independent cohorts.

### Adapting island biogeography theory to the gut

**Figure 3:**
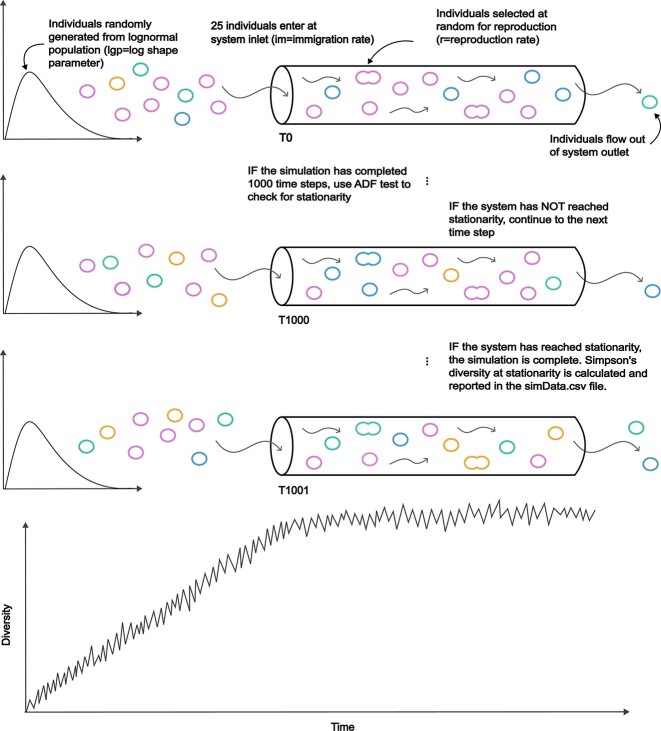
Schematic of the individual-based model (IBM) used to simulate island biogeography theory (IBT) in the gut. We built a simple IBM that approximated the unidirectional flow of the gut, where we could vary the length of a one-dimensional system. Individuals were randomly generated from a heavy-tailed species abundance distribution, entering the system on one side, flowing along the length of the system over time at a constant rate, and eventually exiting the other side of the system. The number of individuals entering the system per time step was determined by the immigration rate of the simulation (im). In addition to flowing through the system (f), some individuals were randomly selected for reproduction (r) at each time step. Simulations were run until the number of individuals and the number of species reached a steady state, as determined by the augmented Dickey–Fuller (ADF) test, before the diversity metric was calculated for each simulation. ADF tests were performed from T1000 (i.e. the 1000th time step) onward, as depicted here. The bottom diversity vs. time plot shows the initial nonstationary period, and the approach toward stationarity over time.

In order to demonstrate a mechanistic link between body size and gut alpha diversity, we reformulated IBT, adapting it to simulate varying gut lengths instead of island areas (Fig. 3 and Table 2). At each time step, the system iterated through the processes: “immigration,” “flow,” and “reproduction,” in a random order. For the immigration step, individuals were randomly sampled from a mainland (i.e. meta-community) lognormal species abundance distribution, entering the system from the inlet. For the flow step, individuals moved one spatial unit down the simulated system (i.e. upstream to downstream; flow was unidirectional). For the reproduction step, all individuals had the same random chance of reproducing, with a probability defined by the reproduction rate. Simulations progressed through these steps in a random order until the simulation had iterated through at least 1000 time steps, at which point the system checked to see if the number of species and individuals had reached stationarity using an Augmented Dickey–Fuller (ADF) test run on the prior 1000 time steps. If the system had not reached stationarity, the simulation would continue and check for stationarity at each time step beyond the 1000th. When stationarity of the system was reached, the steady-state Simpson’s diversity for that model was calculated.

**Table 2:** Individual-based model (IBM) simulation parameters.

Parameter	Abbreviation	Description	Value
Log-series parameter	lgp	Determines the shape of the lognormal population from which individuals are generated	0.9999
Immigration rate	im	Determines the number of individuals entering the system per time step	25
Reproduction	r	Determines the per capita probability of reproduction per time step	0.01
Flow rate	fr	Determines the units of distance each individual flows downstream per time step	1
Length	l	Determines the length of the system	1–1000

The following table lists the parameters in our IBMs and the value(s) of each parameter used in the simulations presented in the main results section.

**Figure 4:**
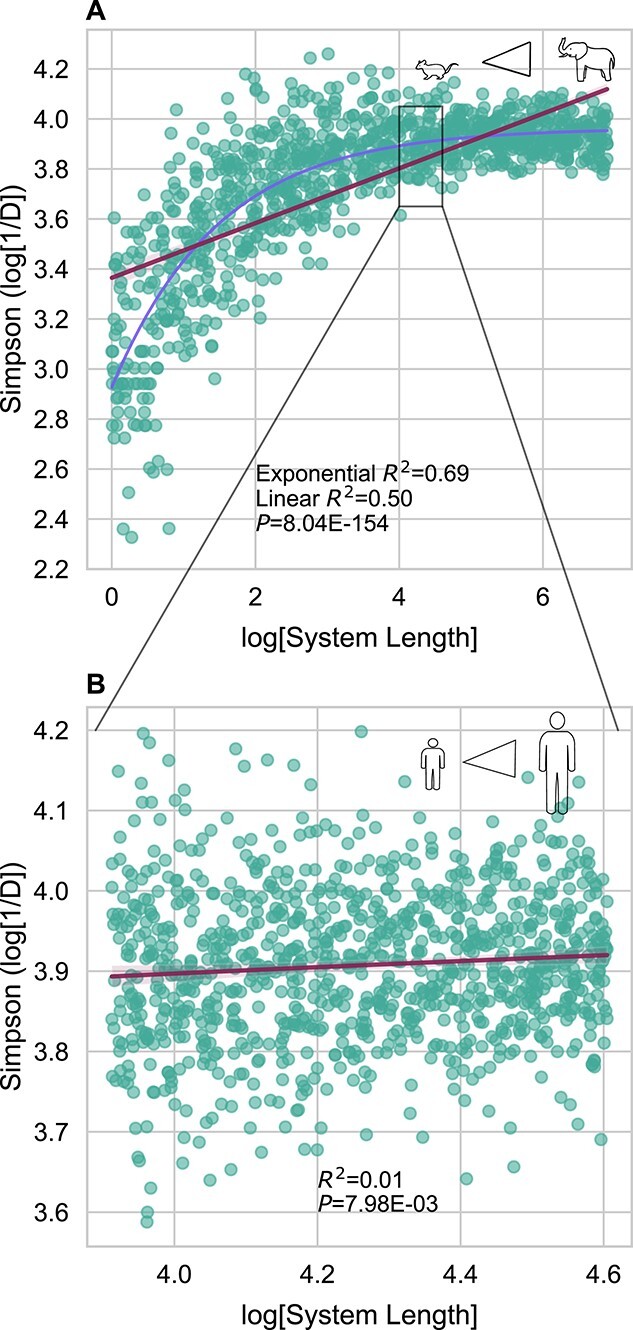
Simulations mirror empirical relationships observed between size and diversity. Both plots show 1000 simulations, where the length of the system was varied within a set range. (A) When varying IBM lengths over three orders of magnitude to approximate vertebrate gut size range, we see a strong association between size and diversity. We find that an exponential model is a better fit than a linear model. (B) When varying IBM length over a much smaller 2-fold range, which is more in line with the observed range in human heights, we see a much weaker association between size and diversity, similar to empirical observations. The zoom-in box drawn in panel (A), which illustrated the human size range, is not to scale, but has been increased in size to improve visibility.

We ran 2 sets of 1000 simulations, one approximating the vertebrate body size range (three orders of magnitude; Fig. 4A) and another approximating the human size range (~2-fold; Fig. 4B). All of our simulations had identical and fixed reproduction and immigration rates (Table 2). Most model parameters were fixed, selected following a series of test simulations (Fig. S1). In our series of test simulations, we tested a combination of different parameter settings for immigration rates and reproduction rates. Low, medium, and high immigration rates were defined as 1, 25, and 100, respectively (i.e. the number of individuals sampled from the meta-community at each time step). For testing different reproduction rates, which determined what percentage of the population would be selected for reproduction, we defined low, medium, and high reproduction rates as 0.1, 1, and 10%. Based on test simulation performance (determined by the variance in alpha diversity explained by system length), we decided to use the medium immigration and medium reproduction rates, respectively. Thus, all simulations had an immigration rate of 25 individuals added to the system per time step and a reproduction rate of 1% per time step. In both sets of simulations, steady-state Simpson’s diversity was positively associated with simulated system length (Fig. 4). OLS regression (log[Simpson (1/*D*)] ~ log[system length]) showed that the simulations run on the vertebrate scale had a much larger *R*^2^ value (*R*^2^ = 0.50, *P* < 10^−6^) compared to the simulations run on the human scale (*R*^2^ = 0.01 ∙ 10^−3^, *P* = 7.98 ∙ 10^−3^). Given an apparent saturation effect observed over the larger size ranges, we fit an exponential model, which yielded a higher *R*^2^ (0.69), indicating that over larger size ranges an exponential model is a better fit than a linear model. Overall, we found our simulated data recapitulated the empirically observed positive scaling between body mass, height, and Simpson’s diversity (Figs. 1–2).

### Exploring the clinical implications of the height–diversity relationship in the American gut cohort

**Figure 5:**
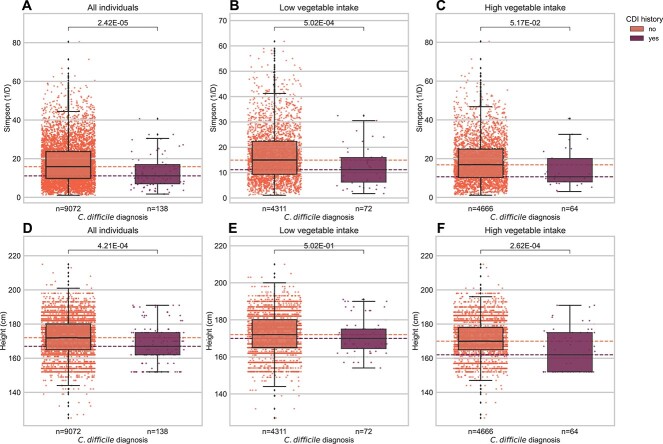
Exploring interactions between height, gut Simpson’s diversity, diet, and self-reported history of CDI in the American Gut cohort. For all plots, dashed lines represent the median of each group, which are shaded by CDI history according to the legend. (A) Individuals who have reported a history of CDI have a significantly lower mean Simpson’s diversity (*n* = 9210) than individuals with a history of CDI (*n* = 138). (B) Of individuals who reported low vegetable intake (*n* = 4311), mean Simpson’s diversity was significantly lower in individuals who had a history of CDI (*n* = 72). (C) Individuals who reported higher vegetable intake (*n* = 4666) did not show significantly lower Simpson’s diversity among those who reported a history of CDI (*n* = 64). (D) The mean height of individuals who reported a history of CDI was significantly lower than individuals who did not. (E) There were no significant differences in mean height between people with and without a history of CDI if they had low vegetable intake. (F) Individuals with a history of CDI were significantly shorter than those who did not among individuals with high vegetable intake. Brackets with stars indicate various levels of significance when performing a Welch’s *t*-test with Bonferroni correction.

Low gut alpha diversity has been associated with susceptibility to enteric infections [8]. We hypothesized shorter individuals, with slightly lower alpha diversity, were perhaps slightly more susceptible to CDI. The American Gut cohort contained individuals with self-reported histories of CDI, which allowed us to explore this hypothesis*.* Consistent with prior literature, individuals who reported a history of CDI (*n* = 138) had less diverse gut microbiomes than those who did not (*n* = 9072; Welch’s *t*-test with Bonferroni correction *t* = 4.76, *P* = 2.42 ∙ 10^−5^; Fig. 5A). Moreover, individuals who reported a history of CDI had a shorter average height than those who did not (Welch’s *t*-test with Bonferroni correction *t* = 3.92, *P* = 4.21 ∙ 10^−4^; Fig. 5D).

We hypothesized that this association between height, diversity, and CDI could be influenced by diet, as prior research has shown that increasing consumption of a larger variety of plants is positively associated with gut microbiome alpha diversity, and more specifically, eating fewer vegetables has been found to be a risk factor for CDI [69, 70]. We partitioned our analysis by self-reported vegetable consumption, looking at differences in height between those with or without a history of CDI in high and low vegetable consumption groups. Of the individuals who reported low vegetable intake, the mean height of individuals with (*n* = 72) and without a history of CDI (*n* = 4311) was not significantly different (Fig. 5E, Welch’s *t*-test with Bonferroni correction *P* = .502). However, of the individuals who reported high vegetable intake, the mean heights of individuals who reported a history of CDI (*n* = 64) were significantly shorter than those who had not (*n* = 4666; Fig. 5F; Welch’s *t*-test with Bonferroni correction *t* = 4.19, *P* = 2.62 ∙ 10^−4^). This variable result across vegetable consumption groups is consistent with the observed shift in the scaling relationship between height and diversity across different dietary contexts (Figs. 1–2). Specifically, we might expect the height effect to be dampened in the low vegetable group if most of the population is unlikely to cross the alpha-diversity threshold for lower CDI risk, whereas this lower risk threshold may be crossed for a larger proportion of the population (i.e. for shorter individuals) in the high vegetable consumption group (Fig. S2). In addition, the observed loss of significance in the low vegetable consumption group could be due to a simple lack of statistical power to detect a relatively weak effect when subsetting the population. Gut alpha diversity tended to be lower in individuals with a history of CDI in both vegetable consumption groups, although the association was on the edge of our significance threshold for the high vegetable consumption group (Fig. 5B and 5C, Welch’s *t*-test with Bonferroni correction *t* = 4.08, *P* = 5.02 ∙ 10^−4^; *t* = 2.51, *P* = .0517, for low and high vegetable intake, respectively).

**Figure 6:**
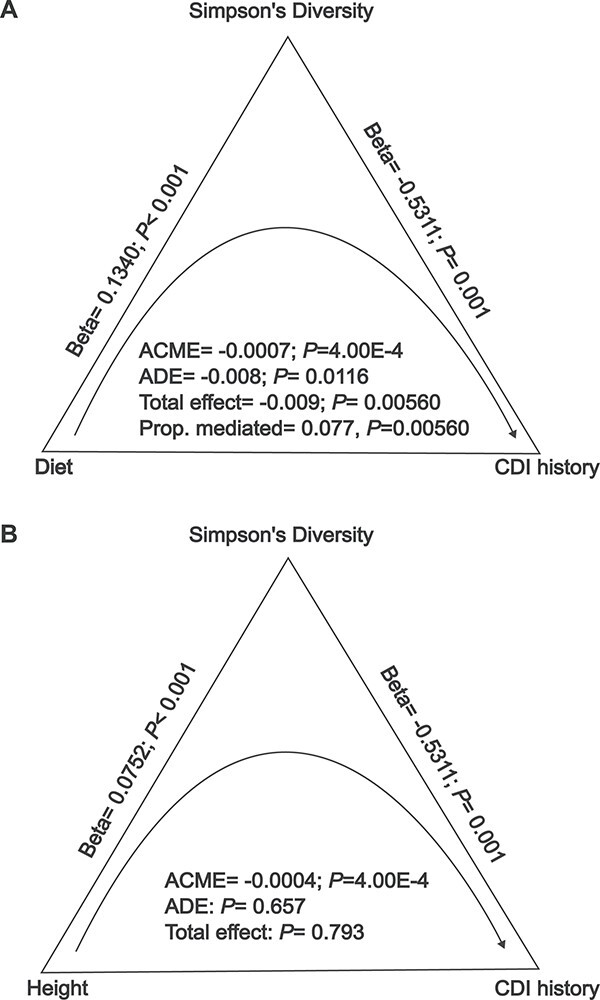
American gut cohort mediation analysis: Simpson’s diversity mediates the impact of diet and height on CDI history. All regressions reported include the covariates: age, sex, BMI, diet (not a covariate in the diet mediation analysis), and BMF. (A) Mediation analysis assigning vegetable consumption as a treatment, Simpson’s diversity as a mediator, and CDI history as an outcome showed that the ACME, ADE, and total effect coefficients were negative and statistically significant (*P* < .05). (B) Mediation analysis assigning height as a treatment, Simpson’s diversity as a mediator, and reported CDI history as an outcome found that only the ACME was statistically significant (*P* < .05). Both diet and height were significantly, positively associated with Simpson’s diversity (*P* < .05). Simpson’s diversity was significantly negatively associated with CDI history (*P* < .05).

We ran a mediation analysis, with bootstrapping (*n* = 5001), to investigate whether the associations between diet, height, and CDI history were mediated by alpha diversity (i.e. testing the niche saturation hypothesis). When we classified vegetable consumption as a treatment, Simpson’s diversity as a mediator, and CDI history as an outcome, we found that the average causal mediated effect (ACME), the average direct effect (ADE), and total effect were all statistically significant (*P* < 4.00 ∙ 10^−4^, *P* = .0116, *P* = .00560, respectively; Fig. 6A). We found evidence for partial mediation, with 8% of the effect of vegetable consumption on CDI history mediated by Simpson’s diversity (Fig. 6A; mediation fraction *P* = .00560). We next classified height as a treatment, Simpson’s diversity as a mediator, and CDI history as an outcome. In the height mediation analysis, the ACME was negative and statistically significant (ACME = −0.0004, *P* = 4.00 ∙ 10^−4^), but the ADE and the total effect were not statistically significant (Fig. 6B; *P* = .657 and *P* = .793, respectively). These results suggest that whatever effect that height may have on CDI history may be completely mediated by Simpson’s diversity.

## Discussion

### Bigger animals harbor more bacterial taxa in their guts

Prior literature has shown that there are many drivers of alpha diversity in the gut microbiota of vertebrates, including gut morphology, evolutionary history, and diet [71–73]. For example, foregut and hindgut fermenters have been shown to have higher gut microbial richness, measured by Shannon diversity, than carnivores with simpler gut anatomies [71]. We found that host body size was strongly associated with gut alpha diversity across several datasets, even when controlling for the host diet (Fig. 1), which suggests that mechanisms independent of gut morphology, phylogeny, and diet are at play.

### Human height is associated with gut microbiome alpha diversity, independent of relevant covariates

Many factors are known to affect human gut microbiome alpha diversity [9, 15, 74, 75]. For example, prior work in less-than-healthy older individuals has shown a decline in alpha diversity with age [76], whereas other studies in healthier older people and in community-dwelling centenarians have shown a decline in core taxa and increased alpha diversity with age [59, 74, 77]. In addition to age, sex has been associated with gut microbiome diversity, with females tending to show higher gut alpha diversity levels than males [74, 78]. Obesity and BMI have been negatively associated with gut alpha diversity, perhaps due to lower dietary fiber intake and higher levels of systemic inflammation associated with obesity [75, 79]. BMF is negatively associated with gut alpha diversity, with individuals experiencing constipation showing higher diversity and individuals experiencing diarrhea showing lower diversity [80]. Finally, a diet rich in plant-based substrates has been associated with higher gut alpha diversity, which is often attributed to the complex polysaccharide content of these foods [70, 81]. To complicate matters further, these demographic variables are highly interrelated. For instance, females, on average, exhibit lower BMFs, shorter heights, and higher fruit and vegetable consumption than males, and the cumulative effects of these entanglements on diversity can be difficult to predict [74, 80, 82]. Furthermore, BMI’s negative association with gut microbiome alpha diversity, and positive association with intestinal inflammation (which can cause shortening of the bowel through smooth muscle contractions), pushes against the overall positive scaling we see between body mass and diversity across vertebrates [75, 79]. Thus, we used height, which has been shown to be positively associated with bowel length [83–86], as an obesity/inflammation-independent proxy for measuring body size in humans. Overall, we were able to control for all of these potentially confounding variables in our regressions, and we found that the associations between height and gut alpha diversity were robust to the inclusion of these variables across two large, independent cohorts (Table 1). Given the vast literature confirming several disease states have been associated with gut microbiota dysbiosis [87, 88], we followed up our regression analysis with a health-adjusted regression using the Arivale cohort. Controlling for several health-associated clinical chemistries and excluding individuals who had taken antibiotics within the last 3 months, we found that the association between height and gut diversity retained its significance. Similar to the vertebrate case above, these results suggest that size–diversity scaling is driven by a mechanism that is independent of dietary intake, inflammation, health status, or bowel movement frequency.

### Adapting IBT to simulate guts of varying lengths

We hypothesized that these size–diversity SARs could be explained by IBT, a simple neutral model that shows how bigger islands harbor a larger number of ecologically equivalent species. We built a simple IBM, designed to approximate IBT in the gut, to show that gut length is indeed positively associated with species diversity (Fig. 4). In our gut-adapted model, intestinal transit time served as a proxy for island area. The amount of variance in Simpson’s diversity explained by system length in the IBMs increased with increasing length scales over which our IBMs were simulated, which closely matched what we observed across both vertebrates and human body size scales (Figs. 1–3). The relationship between system length and diversity appeared to behave somewhat asymptotically over the vertebrate body size range (Fig. 4A), which was similar to what we and others have empirically observed (Fig. 1A) [17]. These results provide a plausible mechanism for the observed size–diversity scaling and add to the body of literature showing how simple neutral models can account for macroecological patterns in real-world microbial communities [19, 20, 89].

### Investigating the clinical implications of IBT in humans

A diverse gut microbiota can be a barrier against invasive pathogens, by saturating available metabolic niches [7, 23, 24, 90]. Thus, our results suggest that height may be a weak predictor of enteric pathogen susceptibility, due to its minor influence on alpha diversity. Indeed, we found that, on average, individuals reporting a history of CDI were shorter and had lower gut alpha diversity than those who had no history of CDI (Fig. 5).

In a prior study, CDI patients tended to report consuming fewer vegetables than individuals without CDI, highlighting the role of diet in CDI prevention [69]. Dietary plant intake has a stronger positive influence over diversity than almost any other lifestyle factor in human cohorts [9, 13, 91, 92]. Vegetable intake was, indeed, strongly associated with gut alpha diversity and with CDI history in the American Gut cohort (Table 1 and Fig. 5). However, the interplay between diet and height was more complex than we anticipated, with height showing a significant association with diversity in the high vegetable intake group, but not in the low vegetable intake group. In order to make sense of this result, we postulated that higher vegetable intake shifts height–diversity scaling such that the putative pathogen susceptibility threshold in alpha diversity is crossed at shorter heights than for the low vegetable consumption group (Fig. S2). This shift could account for the lack of association observed between height and CDI history in the low vegetable consumption group, but a lack of statistical power could also explain this result.

A formal mediation analysis on the effect of diet and height on CDI history yielded evidence that alpha diversity mediated the treatment effect of both height (complete mediation) and vegetable consumption (partial mediation; Fig. 6). Classical mediation analysis does not usually consider the significance of the ACME in the absence of a significant direct or total effect, as was the case with height (Fig. 6) [93]. However, in certain scenarios, this conservative approach can miss true mediation effects, such as when the mediator has nearly the same magnitude effect as the total model or when the direct and mediated effects of the treatment on the outcome are of opposite sign [94–96]. In these scenarios, one can use bootstrapping to estimate confidence intervals for the ACME, as we did here [97]. Overall, diet showed a much stronger association with both diversity and CDI history, suggesting that simple lifestyle interventions, like a higher vegetable intake, can have a larger impact on gut diversity and CDI risk than height.

In conclusion, we find a consistent association between body size and gut microbiome alpha diversity across vertebrates and human populations. The association between human height and gut microbiome alpha diversity was reproducible and was robust to the inclusion of several relevant covariates known to influence gut alpha diversity, including age, sex, BMI, BMF, and diet, across two large, independent cohorts. Furthermore, in the Arivale cohort, we find that this height–diversity scaling is independent of recent antibiotic use and blood measures of LDL cholesterol, HbA1C, and CRP. We showed how this macroecological scaling phenomenon could be plausibly explained by a gut-adapted version of IBT, with simulations closely matching empirical observations. Finally, we explored how the relationship between human height and gut alpha diversity is potentially relevant to CDI risk and how dietary patterns, like vegetable intake, may help mitigate this putative risk. The impact of body size on gut alpha diversity was relatively weak over human body size ranges, and the clinical relevance of this work remains tentative until it can be further validated in additional independent cohorts.

## Study limitations

In our regression analyses, we implicitly consider several deterministic explanations for the observed size–diversity scaling, like the fact that herbivores can achieve larger body sizes than carnivores, bowel movement frequency can vary across sexes, and any number of interactions between our demographic and health-related covariates. None of these factors were able to explain away the observed size–diversity scaling. While we find our gut-adapted formulation of IBT to be a plausible neutral mechanism to explain the size–diversity scaling relationships we observe, there is no definitive way to prove a positive [98], and there may well be other mechanisms at play that were not explored here. For example, we were not able to explore the potential for reverse causation of diversity on height in these datasets, but it is possible that early-life diversity (which could, conceivably, be related to later-life diversity that we measure) may itself influence linear growth of infants and the subsequent height of adults. In addition, in our simulated data, we were only able to span three orders of magnitude in size due to computational constraints, whereas vertebrates span six orders of magnitude in body size. However, we do not necessarily expect a 1:1 scaling relationship between gut length and body mass or height, which is consistent with prior literature [65, 99]. Although real-world guts show complex spatial structure, our model is spatially homogeneous (i.e. a one-dimensional tube). Food enters the mouth, largely devoid of gut bacteria, travels unidirectionally through the gastrointestinal tract, and then exits the anus with around 10^11^ gut bacterial cells per gram feces [100]. The mucus layer and its microbial inhabitants are continually shed outwards toward the lumen, becoming integrated into fecal material as it passes [101]. Thus, despite the highly simplified spatial structure of our simulations, we believe that they capture the major features that are important to our neutral model of the gut: immigration, growth, and unidirectional flow. Finally, we must reiterate that we were likely underpowered to detect an association between height and CDI history in the American Gut cohort, which we expected to be a small effect size due to the already subtle influence of height on alpha diversity. The fact that we did see some signal there is interesting, but the clinical significance of this result is preliminary and will need to be validated in follow-up studies.

## Supplementary Material

6_18_24_Supplement_wrae114

S1_wrae114

S2_wrae114

main_wrae114

## References

[1] Martino C , Dilmore AH, Burcham ZM et al. Microbiota succession throughout life from the cradle to the grave. Nat Rev Microbiol 2022;20:707–20. 10.1038/s41579-022-00768-z 35906422 PMC12875531

[2] Sender R , Fuchs S, Milo R. Revised estimates for the number of human and bacteria cells in the body. PLoS Biol 2016;14:e1002533. 10.1371/journal.pbio.1002533 27541692 PMC4991899

[3] Fan Y , Pedersen O. Gut microbiota in human metabolic health and disease. Nat Rev Microbiol 2021;19:55–71. 10.1038/s41579-020-0433-9 32887946

[4] Diener C , Dai CL, Wilmanski T et al. Genome-microbiome interplay provides insight into the determinants of the human blood metabolome. Nat Metab 2022;4:1560–72. 10.1038/s42255-022-00670-1 36357685 PMC9691620

[5] Cox TO, Lundgren P, Nath K et al. Metabolic control by the microbiome. Genome Medicine 2022;14:80. 10.1186/s13073-022-01092-0 35906678 PMC9338551

[6] Khan I , Bai Y, Zha L et al. Mechanism of the gut microbiota colonization resistance and enteric pathogen infection. Front Cell Infect Microbiol 2021;11:716299. 10.3389/fcimb.2021.716299 35004340 PMC8733563

[7] Spragge F , Bakkeren E, Jahn MT et al. Microbiome diversity protects against pathogens by nutrient blocking. Science 2023;382:eadj3502. 10.1126/science.adj3502 38096285 PMC7616675

[8] Pakpour S , Bhanvadia A, Zhu R et al. Identifying predictive features of Clostridium difficile infection recurrence before, during, and after primary antibiotic treatment. Microbiome 2017;5:148. 10.1186/s40168-017-0368-1 29132405 PMC5684761

[9] McDonald D , Hyde E, Debelius JW et al. American gut: an open platform for citizen science microbiome research. mSystems 2018;3:3. 10.1128/mSystems.00031-18 PMC5954204 29795809

[10] Xu Z , Knight R. Dietary effects on human gut microbiome diversity. Br J Nutr 2015;113:S1–5. 10.1017/S0007114514004127 PMC4405705 25498959

[11] Asnicar F , Leeming ER, Dimidi E et al. Blue poo: impact of gut transit time on the gut microbiome using a novel marker. Gut 2021;70:1665–74. 10.1136/gutjnl-2020-323877 33722860 PMC8349893

[12] Yassour M , Vatanen T, Siljander H et al. Natural history of the infant gut microbiome and impact of antibiotic treatment on bacterial strain diversity and stability. Sci Transl Med 2016;8:343ra81. 10.1126/scitranslmed.aad0917 PMC5032909 27306663

[13] Godon J-J , Arulazhagan P, Steyer J-P et al. Vertebrate bacterial gut diversity: size also matters. BMC Ecol 2016;16:12. 10.1186/s12898-016-0071-2 27008566 PMC4804487

[14] Jernigan A , Hestekin C. Capillary electrophoresis single-strand conformational polymorphisms as a method to differentiate algal species. J Anal Methods Chem 2015;2015:1–7. 10.1155/2015/272964 PMC4460240 26101693

[15] Manor O , Dai CL, Kornilov SA et al. Health and disease markers correlate with gut microbiome composition across thousands of people. Nat Commun 2020;11:5206. 10.1038/s41467-020-18871-1 33060586 PMC7562722

[16] Li S-P , Wang P, Chen Y et al. Island biogeography of soil bacteria and fungi: similar patterns, but different mechanisms. ISME J 2020;14:1886–96. 10.1038/s41396-020-0657-8 32341471 PMC7305213

[17] Matthews TJ , Triantis KA, Whittaker RJ. The Species–Area Relationship: Theory and Application. Cambridge University Press, 2020.

[18] Connor EF , McCoy ED. The statistics and biology of the species-area relationship. Am Nat 1979;113:791–833. 10.1086/283438

[19] Sala C , Vitali S, Giampieri E et al. Stochastic neutral modelling of the gut microbiota’s relative species abundance from next generation sequencing data. BMC Bioinformatics 2016;17:179–88. 10.1186/s12859-015-0858-8 26821617 PMC4959352

[20] Burns AR , Stephens WZ, Stagaman K et al. Contribution of neutral processes to the assembly of gut microbial communities in the zebrafish over host development. ISME J 2015;10:655–64. 10.1038/ismej.2015.142 26296066 PMC4817674

[21] MacArthur RH , Wilson EO. The Theory of Island Biogeography. Princeton University Press, Princeton, New Jersey, 2001.

[22] van Werkhoven CH , Ducher A, Berkell M et al. Incidence and predictive biomarkers of Clostridioides difficile infection in hospitalized patients receiving broad-spectrum antibiotics. Nat Commun 2021;12:2240. 10.1038/s41467-021-22269-y 33854064 PMC8046770

[23] Pereira FC , Berry D. Microbial nutrient niches in the gut. Environ Microbiol 2017;19:1366–78. 10.1111/1462-2920.13659 28035742 PMC5412925

[24] Kamada N , Chen GY, Inohara N et al. Control of pathogens and pathobionts by the gut microbiota. Nat Immunol 2013;14:685–90. 10.1038/ni.2608 23778796 PMC4083503

[25] Tomkovich S , Taylor A, King J et al. An osmotic laxative renders mice susceptible to prolonged Clostridioides difficile colonization and hinders clearance. mSphere 2021;6:e0062921. 10.1128/mSphere.00629-21 34585964 PMC8550136

[26] VanInsberghe D , Elsherbini JA, Varian B et al. Diarrhoeal events can trigger long-term Clostridium difficile colonization with recurrent blooms. Nat Microbiol 2020;5:642–50. 10.1038/s41564-020-0668-2 32042128

[27] Bignardi GE . Risk factors for Clostridium difficile infection. J Hosp Infect 1998;40:1–15. 10.1016/S0195-6701(98)90019-6 9777516

[28] Johnson S . Recurrent Clostridium difficile infection: a review of risk factors, treatments, and outcomes. J Inf Secur 2009;58:403–10. 10.1016/j.jinf.2009.03.010 19394704

[29] Pérez-Cobas A , Moya A, Gosalbes M et al. Colonization resistance of the gut microbiota against Clostridium difficile. Antibiotics 2015;4:337–57. 10.3390/antibiotics4030337 27025628 PMC4790290

[30] Song SJ , Sanders JG, Delsuc F et al. Comparative analyses of vertebrate gut microbiomes reveal convergence between birds and bats. MBio 2020;11:11. 10.1128/mBio.02901-19 PMC6946802 31911491

[31] Groussin M , Mazel F, Sanders JG et al. Unraveling the processes shaping mammalian gut microbiomes over evolutionary time. Nat Commun 2017;8:14319. 10.1038/ncomms14319 28230052 PMC5331214

[32] Liu Q , Scaringe WA, Sommer SS. Discrete mobility of single-stranded DNA in non-denaturing gel electrophoresis. Nucleic Acids Res 2000;28:940–3. 10.1093/nar/28.4.940 10648786 PMC102567

[33] Herberstein ME , McLean DJ, Lowe E et al. Animal traits - a curated animal trait database for body mass, metabolic rate and brain size. Scientific Data 2022;9:1–11. 10.1038/s41597-022-01364-9 35654905 PMC9163144

[34] Laursen L , Bekoff M. Loxodonta Africana. Mammalian Species 1978, 1–8. 10.2307/3503889

[35] Attias N , Gurarie E, Fagan WF et al. Ecology and social biology of the southern three-banded armadillo (Tolypeutes matacus; Cingulata: Chlamyphoridae). J Mammal 2020;101:1692–705. 10.1093/jmammal/gyaa117

[36] Fischer J , Higham JP, Alberts SC et al. Insights into the evolution of social systems and species from baboon studies. elife 2019;8:e50989. 10.7554/eLife.50989 31711570 PMC6850771

[37] Réale D , Festa-Bianchet M, Jorgenson JT. Heritability of body mass varies with age and season in wild bighorn sheep. Heredity (Edinb) 1999;83:526–32. 10.1038/sj.hdy.6885430 10620024

[38] Trani MK , Mark Ford W, Chapman BR. The Land Manager’s Guide to Mammals of the South. Nature Conservancy, Durham, North Carolina, 2007.

[39] Bauchot R , Stephan H. DONNEES NOUVELLES Sur L’ENCEPHALISATION des insectivores et des PROSIMIENS. Mammalia 1966;30:160–96. 10.1515/mamm.1966.30.1.160

[40] Kes Hillman-Smith AK , Groves CP. Diceros bicornis. Mammalian Species 1994, 1–8. 10.2307/3504292

[41] New data on the status and distribution of the bush dog (Speothos venaticus): evaluating its quality of protection and directing research efforts. Biol Conserv 2008;141:2494–505. 10.1016/j.biocon.2008.07.010

[42] Kälin N , Martin RD, Genoud M. Basal rate of metabolism and temperature regulation in Goeldi's monkey (Callimico goeldii). Comp Biochem Physiol A Mol Integr Physiol 2003;135:279–90. 10.1016/S1095-6433(03)00077-1 12781828

[43] Benatti HR , Luz HR, Lima DM et al. Morphometric patterns and blood biochemistry of capybaras (Hydrochoerus hydrochaeris) from human-modified landscapes and natural landscapes in Brazil. Vet Sci 2021;8:165. 10.3390/vetsci8080165 34437487 PMC8402786

[44] Reamer LA , Neal Webb SJ, Jones R et al. Validation and utility of a body condition scoring system for chimpanzees (Pan troglodytes). Am J Primatol 2020;82:e23188. 10.1002/ajp.23188 32856319 PMC7685522

[45] Boddy AM , McGowen MR, Sherwood CC et al. Comparative analysis of encephalization in mammals reveals relaxed constraints on anthropoid primate and cetacean brain scaling. J Evol Biol 2012;25:981–94. 10.1111/j.1420-9101.2012.02491.x 22435703

[46] Dawson TJ , Grant TR, Fanning D. Standard metabolism of monotremes and the evolution of homeothermy. Aust J Zool 1979;27:511–5. 10.1071/ZO9790511

[47] Metabolic rates of three gazelle species (Nanger soemmerringii, Gazella gazella, Gazella spekei) adapted to arid habitats. Mamm Biol 2015;80:390–4. 10.1016/j.mambio.2015.05.008

[48] Crile G . A Record of the Body Weight and Certain Organ and Gland Weights of 3690 Animals. Ohio Journal of Science 1940;40:219–60.

[49] Bertelsen MF . Giraffidae. In: Miller RE, Fowler ME (eds.), Fowler’s Zoo and Wild Animal Medicine, Amsterdam: Elsevier, 2015;8:602–10. 10.1016/B978-1-4557-7397-8.00061-X

[50] Wilson DE , Hanlon E. Lemur catta (primates: Lemuridae). Mamm Species 2010;42:58–74.

[51] Sha JCM . Comparative diet and nutrition of frugivorous and folivorous primates at the Singapore zoo. JZAR 2014;2:54–61.

[52] Gerstner K , Liesegang A, Hatt J-M et al. Seasonal body mass changes and feed intake in spectacled bears (Tremarctos ornatus) at Zurich zoo. JZAR 2016;4:121–6.

[53] Cain JW , Krausman PR, Germaine HL. Antidorcas marsupialis. Mamm Species 2004;753:1–7. 10.1644/753

[54] Lurz PWW , Fielding I, Hayssen V. Callosciurus prevostii (Rodentia: Sciuridae). Mamm Species 2017;49:40–50. 10.1093/mspecies/sex004

[55] Hoefs M . The thermoregulatory potential of Ovis horn cores. Can J Zool 2000;78:1419–26. 10.1139/z00-075

[56] Liu L , Megens H-J, Crooijmans RPM et al. The Visayan warty pig (Sus cebifrons) genome provides insight into chromosome evolution and sensory adaptation in pigs. Mol Biol Evol 2022;39:39. 10.1093/molbev/msac110 PMC9178973 35642310

[57] Saarinen J , Cirilli O, Strani F et al. Testing equid body mass estimate equations on modern zebras—with implications to understanding the relationship of body size, diet, and habitats of Equus in the Pleistocene of Europe. Front Ecol Evol 2021;9:622412. 10.3389/fevo.2021.622412

[58] Callahan BJ , McMurdie PJ, Rosen MJ et al. DADA2: high-resolution sample inference from Illumina amplicon data. Nat Methods 2016;13:581–3. 10.1038/nmeth.3869 27214047 PMC4927377

[59] Wilmanski T , Diener C, Rappaport N et al. Gut microbiome pattern reflects healthy ageing and predicts survival in humans. Nat Metab 2021;3:274–86. 10.1038/s42255-021-00348-0 33619379 PMC8169080

[60] Amir A , McDonald D, Navas-Molina JA et al. Deblur rapidly resolves single-nucleotide community sequence patterns. mSystems 2017;2. 10.1128/msystems.00191-16 PMC5340863 28289731

[61] Hubbell SP . The Unified Neutral Theory of Biodiversity and Biogeography. Princeton (NJ): Princeton University Press, 2001.

[62] Preston FW . The commonness and rarity, of species. Ecology 1948;29:254–83. 10.2307/1930989

[63] Doroghazi JR , Buckley DH. Evidence from GC-TRFLP that bacterial communities in soil are lognormally distributed. PLoS One 2008;3:e2910. 10.1371/journal.pone.0002910 18682841 PMC2483420

[64] Dunbar J , Barns SM, Ticknor LO et al. Empirical and theoretical bacterial diversity in four Arizona soils. Appl Environ Microbiol 2002;68:3035–45. 10.1128/AEM.68.6.3035-3045.2002 12039765 PMC123964

[65] Clauss M , Schwarm A, Ortmann S, Steich WJ, Hummel J. A case of non-scaling in mammalian physiology? Body size, digestive capacity, food intake, and ingesta passage in mammalian herbivores. In: Comparative Biochemistry and Physiology Part A: Molecular & Integrative Physiology. Amsterdam: Elsevier, 2007;148:249–65. 10.1016/j.cbpa.2007.05.024 17643330

[66] Seabold S , Perktold J. Statsmodels: Econometric and Statistical Modeling with Python, 2010, 92–6.

[67] Tingley D , Yamamoto T, Hirose K et al. Mediation: R package for causal mediation analysis. J Stat Softw 2014;59:1–38. 10.18637/jss.v059.i0526917999

[68] Mazel F , Guisan A, Parfrey LW. Transmission mode and dispersal traits correlate with host specificity in mammalian gut microbes. Mol Ecol 2023;33:e16862. 10.1111/mec.16862 36786039

[69] Lang V , Gunka K, Ortlepp JR et al. Risk factors of patients with diarrhea for having infection. Front Microbiol 2022;13:840846. 10.3389/fmicb.2022.840846 35359708 PMC8963458

[70] Tomova A , Bukovsky I, Rembert E et al. The effects of vegetarian and vegan diets on gut microbiota. Front Nutr 2019;6:47. 10.3389/fnut.2019.00047 31058160 PMC6478664

[71] Reese AT , Dunn RR. Drivers of microbiome biodiversity: a review of general rules, feces, and ignorance. MBio 2018;9. 10.1128/mbio.01294-18 PMC6069118 30065092

[72] Muegge BD , Kuczynski J, Knights D et al. Diet drives convergence in gut microbiome functions across mammalian phylogeny and within humans. Science 2011;332:970–4. 10.1126/science.1198719 21596990 PMC3303602

[73] Youngblut ND , Reischer GH, Walters W et al. Host diet and evolutionary history explain different aspects of gut microbiome diversity among vertebrate clades. Nat Commun 2019;10:2200. 10.1038/s41467-019-10191-3 31097702 PMC6522487

[74] de la Cuesta-Zuluaga J , Kelley ST, Chen Y et al. Age- and sex-dependent patterns of gut microbial diversity in human adults. mSystems 2019;4:e00261–19. 10.1128/mSystems.00261-19 PMC6517691 31098397

[75] Wilmanski T , Rappaport N, Earls JC et al. Blood metabolome predicts gut microbiome α-diversity in humans. Nat Biotechnol 2019;37:1217–28. 10.1038/s41587-019-0233-9 31477923

[76] Jeffery IB , Lynch DB, O’Toole PW. Composition and temporal stability of the gut microbiota in older persons. ISME J 2016;10:170–82. 10.1038/ismej.2015.88 26090993 PMC4681863

[77] Biagi E , Franceschi C, Rampelli S et al. Gut microbiota and extreme longevity. Curr Biol 2016;26:1480–5. 10.1016/j.cub.2016.04.016 27185560

[78] Wilmanski T , Gibbons SM, Price ND. Healthy aging and the human gut microbiome: why we cannot just turn back the clock. Nat Aging 2022;2:869–71. 10.1038/s43587-022-00294-w 37118282 PMC10155257

[79] Duan M , Wang Y, Zhang Q et al. Characteristics of gut microbiota in people with obesity. PLoS One 2021;16:e0255446. 10.1371/journal.pone.0255446 34375351 PMC8354443

[80] Johnson JP , Diener C, Levine AE et al. Generally-healthy individuals with aberrant bowel movement frequencies show enrichment for microbially-derived blood metabolites associated with reduced kidney function. bioRxiv 2023. 10.1101/2023.03.04.531100

[81] Leeming ER , Johnson AJ, Spector TD et al. Effect of diet on the gut microbiota: rethinking intervention duration. Nutrient*s* 2019;11:2862. 10.3390/nu11122862 31766592 PMC6950569

[82] Kim YS , Unno T, Kim BY et al. Sex differences in gut microbiota. World J Mens Health 2020;38:48–60. 10.5534/wjmh.190009 30929328 PMC6920072

[83] Minko E , Pagano A, Caceres N et al. Human intestinal tract length and relationship with body height (916.4). FASEB J 2014;28:916.4. 10.1096/fasebj.28.1_supplement.916.4

[84] Bekheit M , Ibrahim MY, Tobar W et al. Correlation between the total small bowel length and anthropometric measures in living humans: cross-sectional study. Obes Surg 2020;30:681–6. 10.1007/s11695-019-04238-z 31686382

[85] Tacchino RM . Bowel length: measurement, predictors, and impact on bariatric and metabolic surgery. Surg Obes Relat Dis 2015;11:328–34. 10.1016/j.soard.2014.09.016 25614357

[86] Gore RM . Colonic contour changes in chronic ulcerative colitis: reappraisal of some old concepts. AJR Am J Roentgenol 1992;158:59–61. 10.2214/ajr.158.1.1727359 1727359

[87] Pickard JM , Zeng MY, Caruso R et al. Gut microbiota: role in pathogen colonization, immune responses, and inflammatory disease. Immunol Rev 2017;279:70–89. 10.1111/imr.12567 28856738 PMC5657496

[88] Lynch SV , Pedersen O. The human intestinal microbiome in health and disease. N Engl J Med 2016;375:2369–79. 10.1056/NEJMra1600266 27974040

[89] Hubbell SP . Neutral theory and the evolution of ecological equivalence. Ecology 2006;87:1387–98. 10.1890/0012-9658(2006)87[1387:NTATEO]2.0.CO;2 16869413

[90] Honda K , Littman DR. The microbiome in infectious disease and inflammation. Annu Rev Immunol 2012;30:759–95. 10.1146/annurev-immunol-020711-074937 22224764 PMC4426968

[91] Losasso C , Eckert EM, Mastrorilli E et al. Assessing the influence of vegan, vegetarian and omnivore oriented westernized dietary styles on human gut microbiota: a cross sectional study. Front Microbiol 2018;9:317. 10.3389/fmicb.2018.00317 29556222 PMC5844980

[92] Cronin P , Joyce SA, O’Toole PW et al. Dietary fibre modulates the gut microbiota. Nutrients 2021;13:1655. 10.3390/nu13051655 34068353 PMC8153313

[93] Baron RM , Kenny DA. The moderator-mediator variable distinction in social psychological research: conceptual, strategic, and statistical considerations. J Pers Soc Psychol 1986;51:1173–82. 10.1037/0022-3514.51.6.1173 3806354

[94] MacKinnon DP , Krull JL, Lockwood CM. Equivalence of the mediation, confounding and suppression effect. Prev Sci 2000;1:173–81. 10.1023/A:1026595011371 11523746 PMC2819361

[95] McFatter RM . The use of structural equation models in interpreting regression equations including suppressor and enhancer variables. Appl Psychol Meas 1979;3:123–35. 10.1177/014662167900300113

[96] Judd CM , Kenny DA. Estimating the Effects of Social Intervention. Cambridge University Press, 1981.

[97] Mackinnon DP , Lockwood CM, Williams J. Confidence limits for the indirect effect: distribution of the product and resampling methods. Multivariate Behav Res 2004;39:99–128. 10.1207/s15327906mbr3901_4 20157642 PMC2821115

[98] Popper K . The Logic of Scientific Discovery. London: Routledge, 2005.

[99] Lavin SR , Karasov WH, Ives AR et al. Morphometrics of the avian small intestine compared with that of nonflying mammals: a phylogenetic approach. Physiol Biochem Zool 2008;81:526–50. 10.1086/590395 18754728

[100] Lim JJ , Diener C, Wilson J et al. Growth phase estimation for abundant bacterial populations sampled longitudinally from human stool metagenomes. Nat Commun 2023;14:5682. 10.1038/s41467-023-41424-1 37709733 PMC10502120

[101] Gustafsson JK , Johansson MEV. The role of goblet cells and mucus in intestinal homeostasis. Nat Rev Gastroenterol Hepatol 2022;19:785–803. 10.1038/s41575-022-00675-x 36097076

