# Supplementary material for: Island biogeography theory provides a plausible explanation for why larger vertebrates and taller humans have more diverse gut microbiomes
Source: ISME J. 2024 Jun 21;18(1):wrae114. doi: 10.1093/ismejo/wrae114 (PMC11253425; doi:10.1093/ismejo/wrae114)
Supplement: 6_18_24_Supplement_wrae114 [file 6_18_24_supplement_wrae114.docx]

**Supplement**


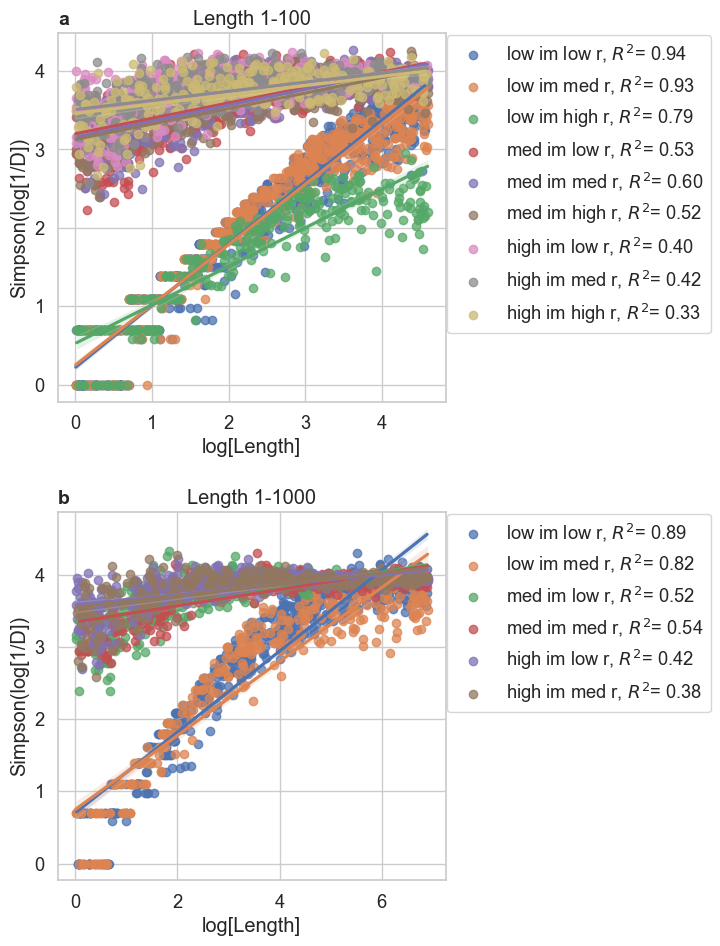


**Figure S1. Testing different immigration and reproduction rate parameters.** Prior to deciding on a set of simulation parameters, we evaluated the effect of different immigration rates and reproduction rates in our IBMs. Low, medium, and high immigration rates (im) were defined as 1, 25, or 50 individuals added to the system per time step. Low, medium and high reproduction rates (r) were defined as 0.001. 0.01, and 0.1 amount of individuals in the population reproducing per time step. As both immigration and reproduction rates increased, we saw a reduction in the variance in Simpson’s Diversity explained by system length. Due to the high computational cost of simulating high reproduction rates in larger systems, we only explored low and medium reproduction rates in panel B. Based on these simulations, we selected a fixed immigration rate of 25 individuals per time step and a fixed reproduction rate of 1% of the population reproducing per time step for the rest of our IBM simulations.


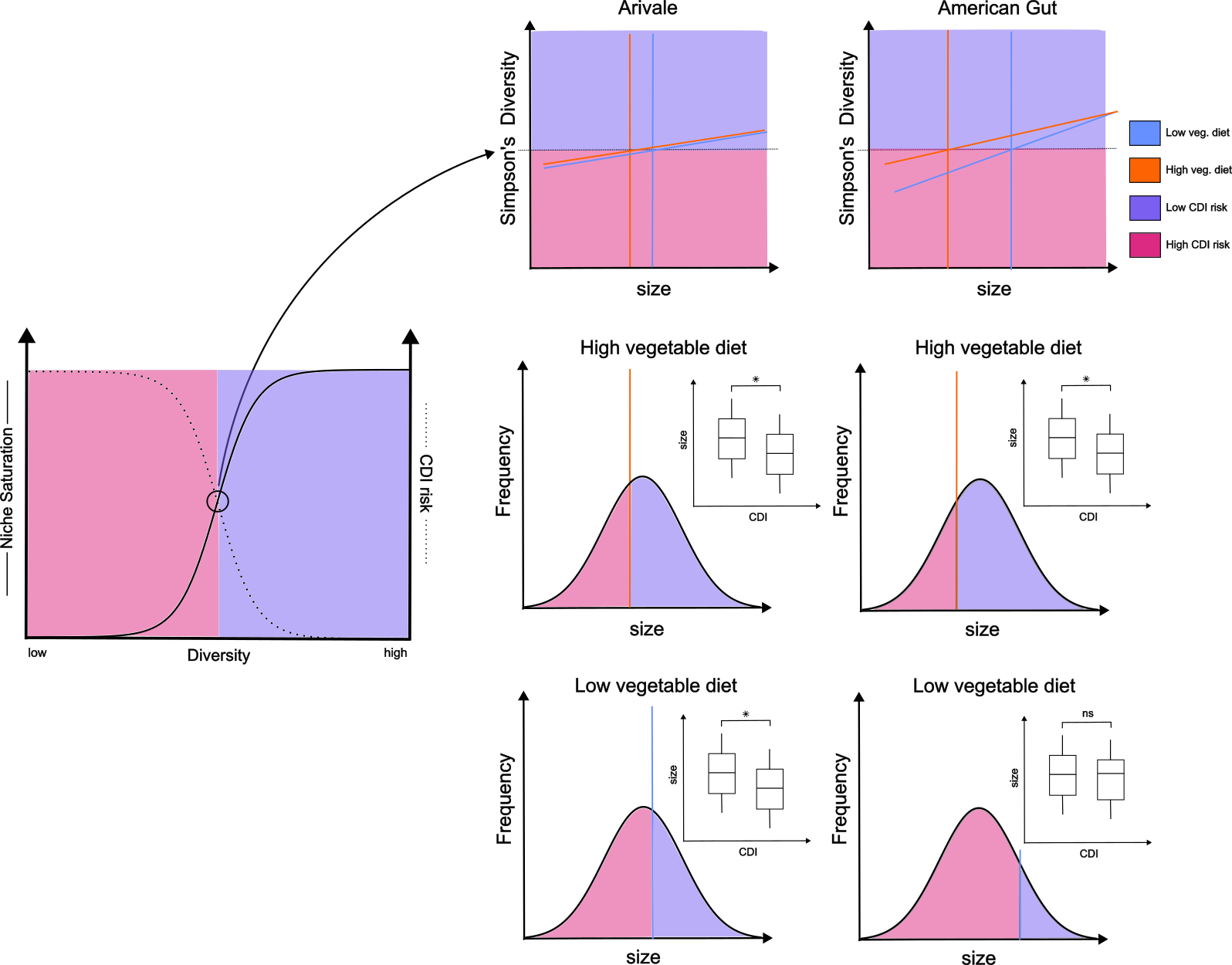


**Figure S2. Understanding the interplay between niche saturation, CDI risk, alpha diversity, height, and diet.** Here we show a possible explanation for the lack of significant differences in mean heights between individuals with and without a history of CDI in the high/low vegetable groups. Assuming a non-linear relationship between CDI susceptibility and gut alpha diversity, where we observe a risk threshold, we expect that individuals with a diet low versus high in vegetables will cross this threshold at different heights. This will give rise to different Ns across risk groups, depending on diet, which we do not have *a priori* knowledge of. These varying risk group Ns will influence our statistical power to detect an association with height (see illustrated examples for Arivale and American Gut cohorts).

**Supplemental File S1**

Python source code to reproduce the simulations in the manuscript.
