# Supplementary figures and images for: Island biogeography theory provides a plausible explanation for why larger vertebrates and taller humans have more diverse gut microbiomes
Source: ISME J. 2024 Jun 21;18(1):wrae114. doi: 10.1093/ismejo/wrae114 (PMC11253425; doi:10.1093/ismejo/wrae114)

**a** Length 1-100

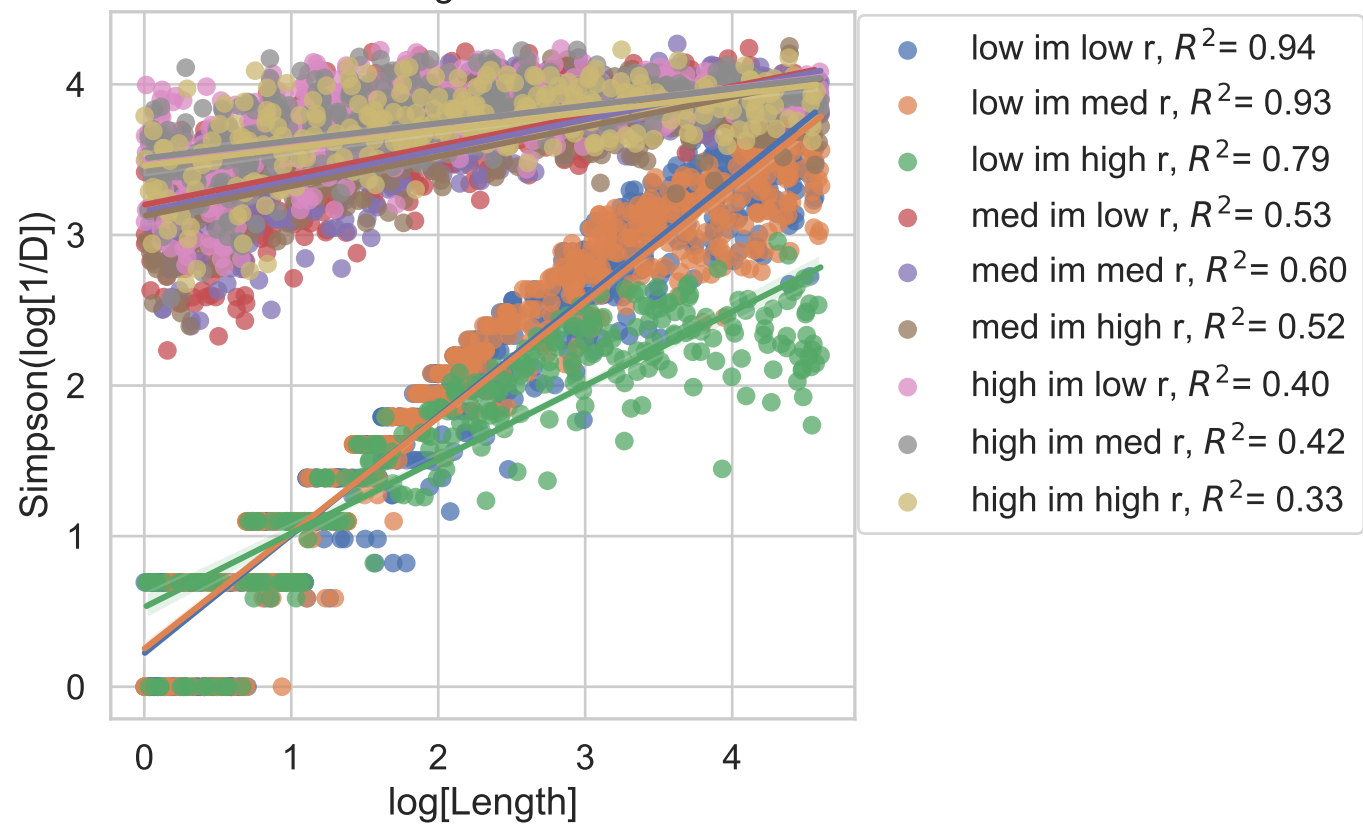

**b** Length 1-1000

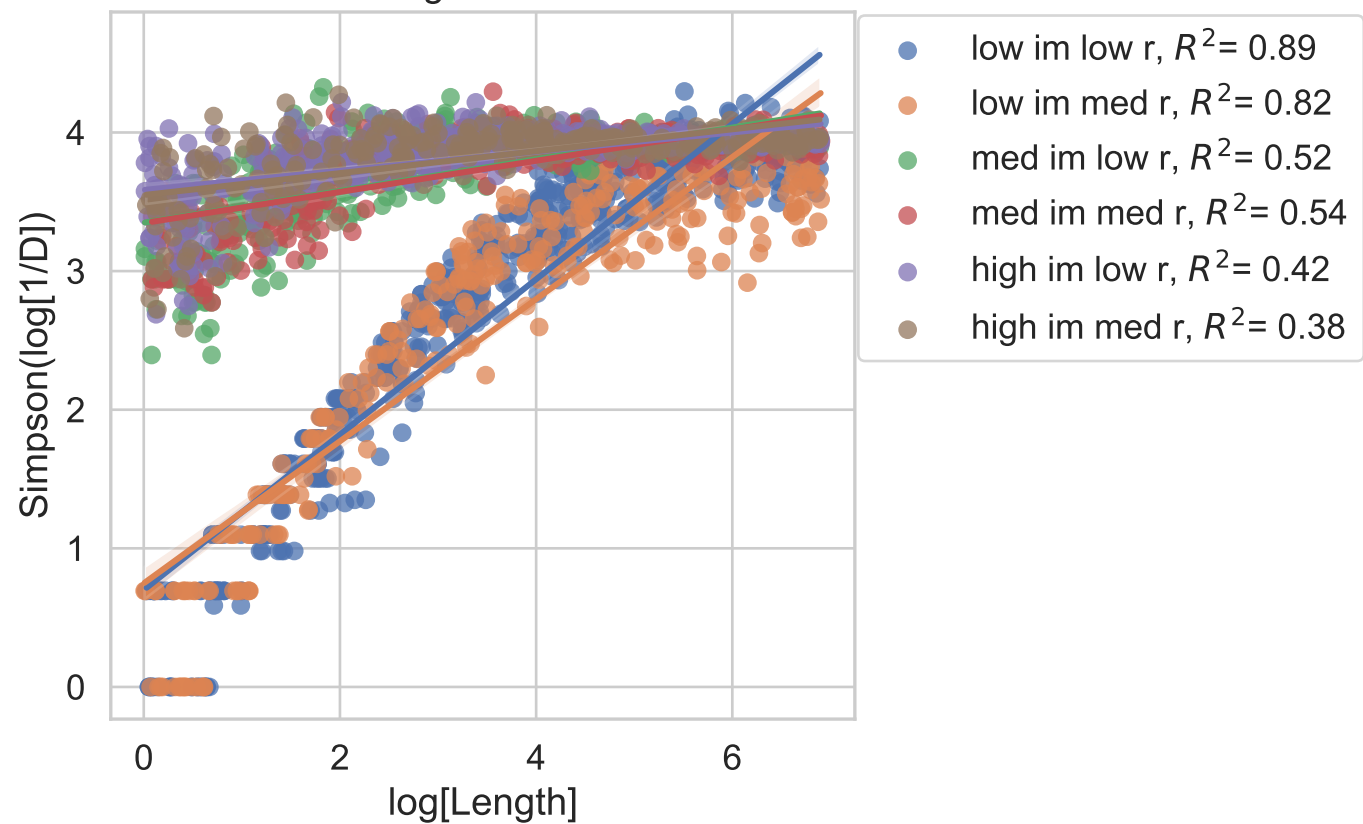

Supplement: S1_wrae114 [file s1_wrae114.pdf]

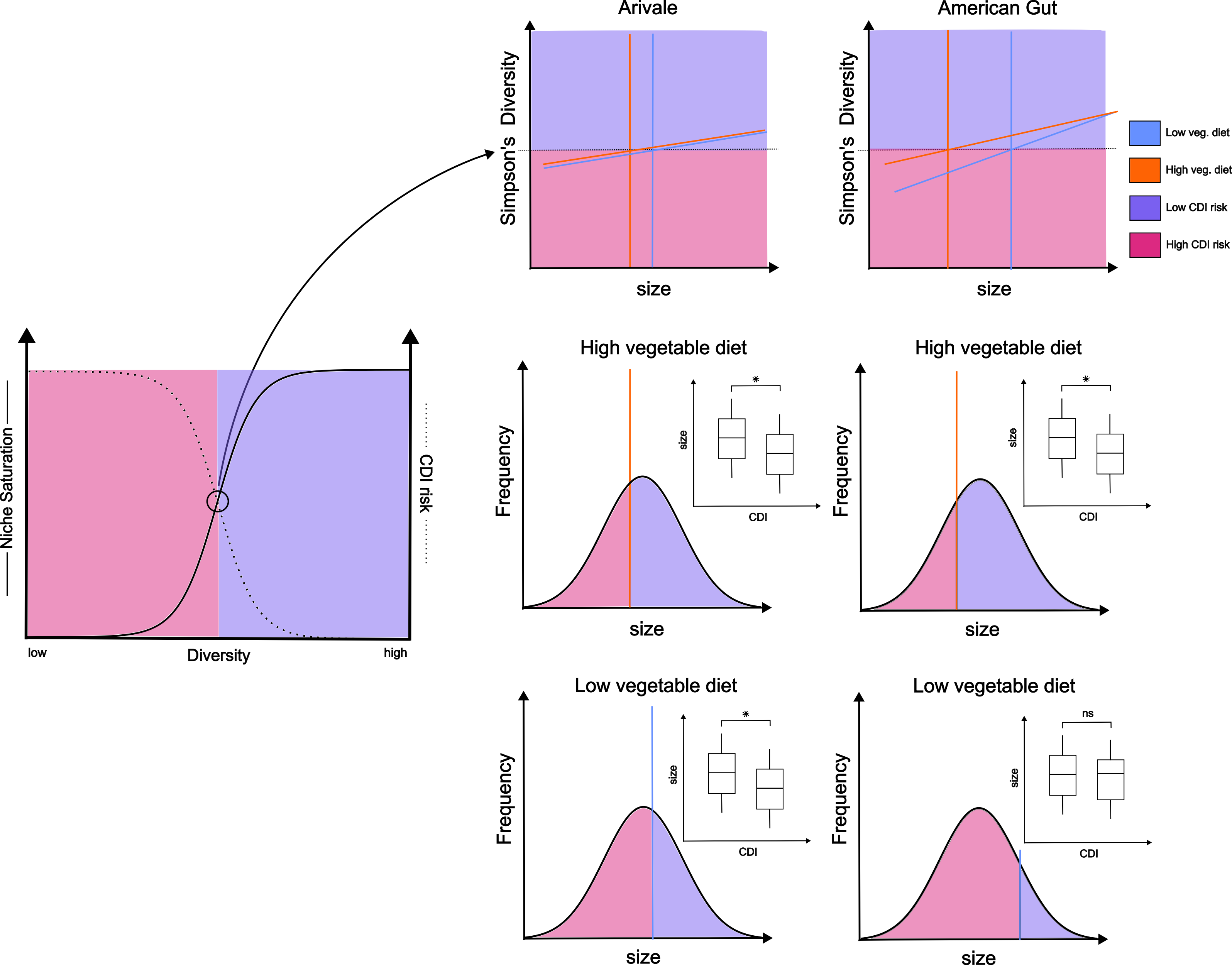

Supplement: S2_wrae114 [file s2_wrae114.pdf]
